# Advanced Botanical Research in the Mediterranean Area: Studies in Honor of Prof. Francesco Maria Raimondo on the Occasion of His 80th Birthday

**DOI:** 10.3390/plants15132067

**Published:** 2026-07-03

**Authors:** Giuseppe Venturella, Gianniantonio Domina

**Affiliations:** Department of Agricultural, Food and Forest Sciences, University of Palermo, Viale delle Scienze, Bldg. 5, 90128 Palermo, Italy

## 1. Introduction

The aim of this Special Issue, entitled “Advanced Botanical Research in the Mediterranean Area: Studies in Honor of Prof. Francesco Maria Raimondo on the Occasion of His 80th Birthday”, was to expand the knowledge on the extraordinary plant diversity of the Mediterranean basin, a global biodiversity hotspot characterized by unique evolutionary lineages, bioclimatic variations, and complex ecological systems that botanical researchers study and preserve. This Special Issue contains 30 scientific articles (23 original research articles, 5 original reviews, 1 Communication, and 1 Essay), contributing to improving the knowledge about this valuable phytogeographical region and that may lead to the development of new strategies for the conservation and sustainable management of the vast amount of threatened species. This Editorial was written to increase the visibility and citation of these studies, celebrating the profound academic legacy of Prof. Raimondo. The classification of these plant communities is based on their geographical location, bioclimatic bio criteria, and geomorphological characteristics. It is also classified according to the historical phytogeographical dynamics, where different evolutionary lineages shape the current Mediterranean landscape.

## 2. Overview of Special Issue

Zacchigna et al. [1] published a research paper entitled “Application of Computer Vision to the Automated Extraction of Metadata from Natural History Specimen Labels: A Case Study on Herbarium Specimens”. Manual metadata extraction from herbarium labels is a slow process limited by traditional OCR systems. This study proposes an end-to-end solution using the multimodal Transformer model Donut-base, which achieved 85% accuracy when tested on a University of Pisa dataset. The main challenges arose from specimens with multiple labels that combined both handwritten and typewritten text. Raimondo [2] describes *Mentha deleoi*, a new species restricted to Isola Grande in the Stagnone of Marsala (NW Sicily). Due to its small population and the negative impacts of historical salt production on its fragile habitat, an urgent in situ and ex situ conservation plan is proposed.

Research in Andalusia reveals 23 *Agave* taxa, with *Agave americana* demonstrating a widespread, aggregated, and heavily human-driven inland distribution. The study highlights that high suitability for the species is linked to anthropized, low-to-intermediate elevation areas, particularly in the Guadalquivir valley and coastal zones [3].

Morphometric analysis of 18th–19th century rose prickles from the 12th-century Almunia del Castillejo de Monteagudo in Spain identifies *Rosa moschata* and *Rosa foetida* [4]. The findings indicate a continuity of cultivation for these West Asian species, highlighting the impact of medieval Islamic horticulture in al-Andalus gardens.

Di Gristina et al. [5] present a study on the ornamental vascular flora of Basilicata that records 281 taxa, showing a dominance of phanerophytes primarily native to Asia and America. The findings reveal that alien species make up approximately 80% of the region’s urban greenery, reflecting a global trend in landscape homogenization. In a second paper [6], a checklist of 928 ornamental vascular plant taxa found in Sicily’s public and private green spaces was presented. It details each taxon’s origin, growth form, and status, noting a high prevalence of Asiatic, American, and tropical species. Notably, 41% of the documented flora consists of alien species, with *Delonix regia* unique to Sicilian open-air cultivation. In a third publication on urban ornamental flora, Venturella et al. [7] fill a critical knowledge gap by presenting a comprehensive census of 287 ornamental tree, shrub, and succulent taxa recorded across six Apulian provinces between 2021 and 2024. It details their distribution, growth forms, and geographic origins, revealing a high presence of non-native species. Notably, over 51% of the surveyed flora consists of alien taxa, with a marked predominance of casual and naturalized neophytes.

An evaluation of the Italian National Recovery and Resilience Plan forest restoration measure shows successful implementation across metropolitan cities, with over 4 million native trees and shrubs planted across 4000 hectares. Adhering to strict ecological criteria, the model has exceeded expectations in supporting biodiversity, offering a best practice for urban ecosystem recovery [8].

*Origanum majorana* stems exhibit phytochemical profiles, antioxidant capacity, and cardioprotective effects like the plant’s leaves and flowers, presenting a sustainable source for bioactive compounds. While the stems show slightly lower anti-inflammatory and cytotoxic activities, their functionality supports potential reuse in nutraceutical applications [9].

The study of Poponessi et al. [10] provides an updated analysis of bryophyte diversity on small Italian, Sicilian, and Sardinian islands, highlighting the impact of geographical features such as area, altitude, and substrate on species composition. It examines how these environmental variables, along with ecological indicators, shape the functional traits of Mediterranean bryophyte floras.

Clò et al. [11] analyzed pollen data from 14 archeological sites in southern Italy using the BRAIN database to reconstruct past plant diversity and land management. The research validates historical flora data from Campania, Basilicata, and Sicily, highlighting ubiquitous pollen taxa to better understand human–environment relationships across time.

A study in the area surrounding Tirli, Tuscany, identified 128 wild edible plant taxa, highlighting a rich floristic diversity dominated by Asteraceae and confirming significant correlations between specific life-forms and edible parts. Biochemical analysis validated the nutritional value and antioxidant properties of these plants, supporting the preservation of both local biodiversity and traditional culinary heritage [12].

González et al. [13] presented an ethnobotanical study conducted in Ibiza from 2016 to 2023 with the identification of 254 plant taxa across 71 botanical families, with Solanaceae and Fabaceae being the most frequently cited. Based on 95 interviews with local informants, the research demonstrates that the island’s population still retains significant traditional knowledge regarding local plant biodiversity. Cáceres et al. [14] analyzed traditional medicinal plant knowledge within the Catalan linguistic area, compiling 15,252 reports on 630 taxa used to treat gastrointestinal, metabolic, and nutritional disorders. The research shows a high consensus among informants, with *Matricaria recutita*, *Thymus vulgaris*, and *Lippia triphylla* emerging as the most reported species, primarily administered as tisanes. Notably, 57.59% of these traditional gastrointestinal and metabolic uses were validated by the existing pharmacological literature.

Smeriglio et al. [15] analyzed the micro-morphological, phytochemical, and biological features of leaves from *Ptilostemon greuteri*, a rare Sicilian paleoendemic species. Chemical profiling identified specific compounds for its essential oil, n-hexane, and hydroalcoholic extracts. In vitro assays revealed that the hydroalcoholic extract has the highest antioxidant activity, the essential oil offers strong anti-inflammatory properties, and the n-hexane extract provides the best protease inhibitory effect.

Campisi and Marino [16] analyzed 224 bryophyte taxa from Sicilian aquatic and humid habitats, representing 36% of the island’s total bryological flora across 55 families. It evaluates their biological, ecological, and chorological traits, highlighting specific patterns of vulnerability and resilience to climate change. The findings emphasize the need for future monitoring, particularly for conservation-priority and threatened species in Italy and Europe.

Capaci et al. [17] established the first bioreactor-based micropropagation protocol for the endangered Sicilian endemic *Viola ucriana* using the RITA^®^ temporary immersion system. The TIS method, combined with specific plant growth regulators and rockwool rooting, significantly outperformed conventional solid culture media. Flow cytometry confirmed the genetic fidelity of the regenerated plantlets, offering a highly efficient method with over a 90% acclimatization rate for ex situ conservation.

Seggi et al. [18] provide a practical guideline for optimizing the digitization of small, historical Natural History Collections by focusing on cost- and time-effective pre-digitization planning. Using an image-to-data workflow and the Darwin Core standard scheme, the authors successfully digitized the historical Vatova and Schiffner algarium. The resulting open-access metadata and digital images enhance the collection’s accessibility and preservation while showcasing how standardized protocols advance natural history heritage.

Malfa et al. [19] evaluate the cosmeceutical potential of *Sulla coronaria* flower extract by analyzing its phytochemical profile and biological activity on human dermal fibroblasts. The extract showed significant phenolic content—particularly rutin, quercetin, and isorhamnetin derivatives—yielding strong antioxidant and safe anti-inflammatory effects against interleukin-induced inflammation. These findings confirm its viability as a valuable bioactive source for skincare and nutraceutical applications.

A study analyzes the syntaxonomical, ecological, and conservation features of different European habitats (peat vegetation, such as mires, swamp mires, fens, and peat bogs) on the mountain plateaus of the Central Apennines. These rare Mediterranean relic vegetation fragments are poorer in northern diagnostic species than Central and Northern European communities but rich in surrounding pasture flora, leading to the description of a new subassociation (*Caricetum davallianae caricetosum hostianae*). The findings highlight that these climate-threatened habitats require urgent monitoring due to their vulnerability to global warming and anthropogenic pressures [20].

Greuter [21] presented a catalog of the 339-sheet type herbarium from the Herbarium Greuter in Palermo, focusing predominantly on Mediterranean, Caribbean, and Australian specimens. The collection includes transcribed labels, links to digital images, and protologue texts. Additionally, the paper introduces new nomenclatural terminology, specifically “first-step holotype” and “second-step holotype,” alongside the clarifying phrase “detailed here” for second-step typifications.

Domina et al. [22] identified 137 poisonous and allergenic plant taxa across 100 Sicilian parks and gardens, primarily belonging to the *Solanaceae*, *Moraceae*, *Apocynaceae*, and *Fabaceae* families. Many of these recorded plants cause severe gastrointestinal issues, with 21 identified as deadly poisonous to humans and pets. The findings highlight the need for safer urban garden management strategies and increased public environmental education to balance biodiversity preservation with public safety.

Rocchi et al. [23] provide the first evidence of how simulated gastrointestinal digestion impacts the polyphenols and antioxidant activity of wild versus micropropagated Apennines Genepì infusions. Although wild infusions exhibited superior phenolic levels and antioxidant potential before digestion, these differences significantly narrowed post-digestion due to complex chemical transformations and rearrangements. These findings offer crucial insights into how Genepì infusions might behave and impact human health upon consumption.

Caneva et al. [24] provide the first comprehensive botanical analysis of recurring plant patterns in 6th–4th century BCE Achaemenid art at Persepolis and Susa, linking them to Zoroastrian religious symbolism and medicinal or psychotropic attributes. The research identifies traditional symbols like palms and rosettes, while introducing newly recognized taxa in Persian iconography, such as *Mandragora officinalis* and *Pinus brutia*. These botanical motifs served as profound symbols of power, fertility, immortality, and divine connection, reflecting strong Mesopotamian and Egyptian cultural influences.

The review by Kozuharova et al. [25] analyzes 167 edible wild Asteraceae species traditionally consumed across 11 Mediterranean countries, highlighting a strong divergence between natural plant distribution and actual culinary use. Spain, southern Italy, and Morocco reported the highest numbers of consumed species, yet 106 plants are eaten in only one specific country despite growing in neighboring regions. This indicates that while climate creates shared distribution ranges, ethnobotanical knowledge remains highly localized with limited cross-border transmission.

The review by Bajona et al. [26] highlights that knowledge regarding the 220 upland mire sites in the Italian Apennines, Sicily, and Sardinia is critically outdated, with most historical sites left unsurveyed for over a decade. Concentrated mainly in the Tuscan-Emilian Apennines, Sicily, and Calabria, these fragmented peatland habitats face severe threats from uncontrolled grazing, groundwater extraction, and infrastructure development. The authors emphasize that urgent new field investigations are essential to update biodiversity data and establish effective national and European conservation strategies.

The comprehensive review by Tocci et al. [27] synthesizes the scientific literature up to March 2025 on *Hypericum hircinum*, a Mediterranean medicinal plant traditionally used for respiratory, wound, and pain management. It outlines the species’ diverse phytochemical profile—rich in terpenes, phenolic acids, flavonoids, and phloroglucinols—alongside a wide range of confirmed pharmacological actions, including antimicrobial, antidepressant, and antioxidant activities. These consolidated findings deepen the understanding of the species’ biological value and highlight its potential for future therapeutic applications.

The review by Kozuharova et al. [28] analyzes 81 wild Apiaceae species traditionally consumed as food across 11 Mediterranean and adjacent countries, mapping their natural distribution against actual culinary use. It reveals that 50 taxa are eaten in only one specific country despite growing in neighboring regions, demonstrating that ethnobotanical food traditions remain highly localized. Statistical analyses, including the Jaccard index and heatmap clustering, highlight that local climate factors often influence wild plant consumption more than shared borders or cultural interactions.

The review by Valdés [29] challenges the long-held belief that entomogamy is the sole pollination mechanism in the Mediterranean by demonstrating that ornithogamy and saurogamy also occur. It highlights that primarily insectivorous passerine birds, such as *Sylvia atricapilla*, contribute to pollinating species like *Anagyris foetida*, while the lacertid lizard *Podarcis lilfordi* acts as an effective pollinator on western Mediterranean islands for plants like *Euphorbia dendroides*.

Finally, the Essay by Nualart et al. [30] highlights how climate warming and human activities like tourism are increasing the vulnerability of mountain ecosystems to biological invasions, allowing alien plants to spread rapidly upslope. In the Pyrenees, a Mediterranean biodiversity hotspot with high endemism, cataloging efforts revealed 771 alien taxa, surpassing figures for larger ranges like the Alps. These findings challenge traditional assumptions about mountain resilience and underscore the urgent need for coordinated monitoring and management strategies to protect these vital glacial refugia.

## 3. Conclusions

Overall, the 30 papers gathered in this Special Issue demonstrate how Mediterranean botanical research is addressing modern ecological challenges through a perfect mix of tradition and technological innovation. On one hand, the integration of computer vision applied to herbaria opens innovative scenarios for the digitalization of museum data, while on the other hand, the finding of new endemic species like *Mentha deleoi* and the historical study of tree introductions in al-Andalus confirm the unrelieved dynamism of systematics and phytogeography. Additionally, the data emerging from urban greenery in Basilicata, Sicily, and Apulia highlight a strong and worrying landscape homogenisation characterized by a heavy presence of alien species, which demands urgent sustainable management strategies. In concert, the success of national reforestation plans and the valorisation of floristic waste for nutraceutical purposes pave the way for a concrete circular bioeconomy. Lastly, the biogeographical analyses on minor islands and archeological pollen reconstructions offer fundamental predictive tools to understand biodiversity evolution and the resilience of fragile ecosystems in the face of global climate change.

## Data Availability

No new data were created or analyzed in this study. Data sharing is not applicable to this article.
